# Supplementary material for: Deeply conserved and expressed small ORFs in the model beetle Tribolium castaneum
Source: BMC Biol. 2026 Jul 28;24:218. doi: 10.1186/s12915-026-02686-5 (PMC13670568; doi:10.1186/s12915-026-02686-5)
Supplement: Supplementary file 1 [file 12915_2026_2686_MOESM4_ESM.pdf]

ADDITIONAL FILE 2. Supplementary tables and search commands.

Table S1. GC content per codon in *T. castaneum*.

	GC content			Average percentage
	First base	Second base	Third base	
Putative small ORFs	45.13%	42.88%	46.58%	44.87%
Random small ORFs	40.68%	43.52%	46.95%	43.72%
Tribolium genomic CDSs	49.52%	38.14%	51.09%	46.25%

Table S2. GC content per codon of putative small ORFs in *T. castaneum*, categorized by their conservation levels.

	GC content			Average percentage
	First base	Second base	Third base	
Tribolium	45.13%	42.88%	46.58%	44.87%
Coleoptera	46.07%	46.02%	49.20%	47.10%
Insecta	46.65%	44.63%	50.27%	47.18%
Hexapoda	47.87%	42.91%	53.78%	48.19%
Pancrustcea	48.43%	43.62%	54.42%	48.82%
Arthropoda	46.45%	40.90%	51.81%	46.39%
Ecdysozoa	47.54%	41.28%	52.49%	47.10%
Protostomia	48.36%	43.87%	50.93%	47.72%
Eubilateria	46.91%	42.68%	49.99%	46.53%
Neuralia	46.32%	42.20%	49.64%	46.05%
Eumetazoa	47.14%	43.30%	54.27%	48.24%
Metazoa	47.90%	39.12%	52.96%	46.66%
Opisthokonta	49.57%	41.92%	52.31%	47.93%
Amorphea	48.59%	43.70%	53.38%	48.56%
Eukarya	48.19%	42.03%	50.17%	46.80%
Neomura	49.90%	48.13%	56.97%	51.67%
Neomura + Bacteria	55.17%	45.81%	50.29%	50.42%

Table S3. Amino acid usage of predicted peptide/protein sequences of ORFs in *T. castaneum*.

Amino acids	Putative small ORFs	Randon small ORFs	Large ORFs
A	54 .43	42 .76	58 .73
V	57 .63	54 .56	63 .69
L	87 .65	97 .68	91 .29
I	58 .72	58 .91	56 .10
P	52 .56	50 .48	54 .92
M	37 .59	60 .49	20 .81
F	47 .92	50 .86	38 .85
W	18 .09	19 .54	10 .83
G	53 .61	44 .88	53 .78
S	81 .02	86 .61	78 .52
T	61 .61	67 .44	60 .02
C	31 .19	33 .36	19 .59
N	45 .58	43 .21	49 .72
Q	36 .15	35 .75	43 .09
Y	29 .79	26 .45	30 .56
D	32 .60	20 .15	53 .08
E	44 .61	27 .13	69 .56
K	65 .47	64 .77	69 .36
R	76 .31	90 .16	53 .32
H	27 .49	24 .81	24 .19

Table S4. Codon relative frequency of the putative small ORFs of *T. castaneum*, categorized by their conservation levels.

		Putative small ORFs of *Tribolium castaneum*															
Codon	Amino acid	Coleoptera	Insecta	Hexapoda	Pancrustacea	Arthropoda	Ecdysozoa	Protostomia	Eubilateria	Neuralia	Eumetazoa	Metazoa	Opisthokonta	Amorphea	Eukarya	Archaea	Bacteria
GCA	A	15 .93	14 .64	16 .95	12 .99	11 .57	8 .13	12 .41	13 .04	12 .31	5 .48	10 .34	6 .27	9 .43	11 .14	11 .79	11 .70
GCC	A	14 .60	16 .67	23 .15	21 .54	20 .43	22 .21	20 .14	18 .45	21 .60	17 .23	19 .53	19 .70	23 .99	19 .15	33 .40	29 .24
GCG	A	12 .49	11 .97	12 .40	11 .40	9 .53	11 .92	11 .51	10 .50	11 .56	13 .31	9 .77	12 .99	12 .00	10 .79	11 .79	5 .85
GCT	A	14 .25	14 .09	14 .06	16 .16	15 .15	17 .34	17 .08	16 .38	16 .08	14 .10	14 .36	21 .05	16 .28	17 .76	23 .58	21 .44
TGC	C	16 .70	16 .85	16 .95	19 .96	17 .70	21 .67	17 .98	16 .70	17 .84	24 .28	17 .81	20 .15	28 .28	18 .45	19 .65	9 .75
TGT	C	17 .05	17 .22	13 .64	15 .52	17 .02	18 .96	17 .98	17 .17	20 .35	22 .71	14 .93	17 .47	22 .28	19 .15	11 .79	17 .54
GAC	D	15 .16	16 .94	19 .84	19 .64	22 .13	18 .96	18 .34	16 .38	16 .33	17 .23	20 .10	18 .81	18 .00	18 .45	7 .86	15 .60
GAT	D	17 .05	17 .59	18 .60	20 .91	20 .09	20 .31	19 .96	18 .76	20 .35	24 .28	23 .55	23 .74	23 .14	21 .59	23 .58	19 .49
GAA	E	26 .10	28 .82	29 .35	26 .61	30 .13	32 .23	30 .03	31 .17	29 .89	24 .28	32 .17	35 .38	31 .71	34 .47	21 .61	35 .09
GAG	E	14 .53	17 .41	19 .43	21 .86	19 .40	19 .50	18 .34	18 .13	17 .84	17 .23	26 .42	19 .70	16 .28	17 .76	11 .79	15 .60
TTC	F	18 .74	18 .23	21 .50	21 .22	22 .13	25 .46	22 .65	21 .47	22 .61	23 .49	19 .53	19 .70	20 .57	20 .54	23 .58	23 .39
TTT	F	24 .28	23 .48	19 .02	21 .22	23 .66	21 .13	21 .94	24 .17	23 .36	18 .79	22 .98	19 .70	14 .57	23 .33	11 .79	9 .75
GGA	G	17 .89	19 .25	14 .06	22 .17	19 .57	22 .48	19 .78	20 .83	21 .35	23 .49	17 .81	19 .26	21 .42	20 .89	23 .58	38 .99
GGC	G	13 .47	14 .37	17 .78	19 .05	15 .15	15 .71	16 .90	13 .68	16 .33	22 .71	12 .64	13 .88	22 .28	17 .76	29 .47	21 .44
GGG	G	13 .96	15 .38	15 .71	17 .11	15 .15	15 .44	17 .44	16 .38	17 .08	22 .71	16 .08	14 .78	18 .00	14 .28	13 .75	9 .75
GGT	G	12 .14	12 .71	10 .34	11 .40	12 .77	14 .90	13 .66	12 .88	13 .31	10 .18	15 .51	12 .09	15 .42	11 .49	11 .79	21 .44
CAC	H	14 .03	14 .37	13 .64	14 .25	16 .17	17 .88	15 .10	15 .11	15 .32	19 .58	13 .79	20 .60	18 .00	18 .45	19 .65	23 .39
CAT	H	12 .98	13 .91	10 .75	9 .50	12 .09	13 .27	15 .46	13 .99	12 .06	14 .10	12 .64	14 .78	14 .57	16 .02	9 .82	13 .65
ATA	I	15 .65	13 .54	11 .99	11 .40	13 .45	11 .92	13 .31	13 .68	12 .81	18 .01	10 .91	10 .30	12 .00	13 .93	7 .86	11 .70
ATC	I	15 .65	16 .48	20 .67	21 .22	19 .92	21 .13	17 .62	18 .92	18 .84	15 .66	18 .96	18 .36	19 .71	16 .71	31 .43	27 .29
ATT	I	22 .95	23 .85	20 .26	22 .49	23 .15	24 .11	24 .81	26 .08	24 .87	21 .14	21 .25	19 .70	21 .42	20 .89	13 .75	11 .70
AAA	K	39 .79	39 .23	44 .23	38 .64	48 .51	46 .32	44 .95	44 .36	49 .99	42 .29	53 .42	45 .68	49 .70	51 .18	37 .33	50 .68
AAG	K	23 .79	25 .79	26 .87	27 .56	24 .68	22 .48	23 .55	25 .28	21 .35	25 .84	26 .42	24 .63	22 .28	21 .24	21 .61	1 .95
CTA	L	9 .40	9 .21	9 .10	7 .29	8 .85	7 .58	9 .53	9 .54	7 .79	6 .27	9 .77	10 .30	6 .00	10 .79	5 .89	3 .90
CTC	L	12 .21	12 .06	13 .64	12 .67	11 .40	11 .65	11 .69	12 .09	11 .30	12 .53	10 .91	12 .54	9 .43	12 .88	31 .43	29 .24
CTG	L	14 .17	15 .29	17 .78	16 .47	12 .43	13 .27	13 .31	12 .88	13 .06	16 .45	14 .36	13 .88	13 .71	11 .14	9 .82	7 .80
CTT	L	12 .98	12 .43	14 .88	11 .09	10 .72	12 .46	12 .41	11 .61	13 .56	10 .18	9 .19	11 .64	9 .43	9 .75	3 .93	13 .65
TTA	L	15 .65	16 .12	12 .82	12 .35	14 .47	13 .27	12 .23	14 .95	15 .32	11 .75	15 .51	12 .99	7 .71	18 .11	7 .86	11 .70
TTG	L	20 .98	22 .65	23 .56	23 .44	24 .17	25 .46	22 .11	23 .06	24 .62	19 .58	27 .00	26 .42	23 .14	20 .20	13 .75	7 .80
ATG	M	35 .93	35 .55	31 .42	32 .63	35 .57	36 .02	33 .44	34 .98	34 .92	37 .59	34 .46	33 .59	36 .85	33 .77	29 .47	23 .39

Codon	Amino acid	Coleoptera	Insecta	Hexapoda	Pancrustacea	Arthropoda	Ecdysozoa	Protostomia	Eubilateria	Neuralia	Eumetazoa	Metazoa	Opisthokonta	Amorphea	Eukarya	Archaea	Bacteria
AAC	N	20.21	20.81	21.91	24.71	23.49	23.84	19.60	21.78	20.35	22.71	24.70	24.63	25.71	22.63	21.61	23.39
AAT	N	20.84	21.55	18.19	18.37	21.79	19.50	17.08	19.56	23.61	19.58	22.40	17.02	19.71	19.50	13.75	9.75
CCA	P	16.98	14.55	14.06	16.16	11.75	9.21	14.20	14.79	11.30	10.18	15.51	13.44	12.85	12.19	7.86	9.75
CCC	P	14.25	16.12	15.71	18.69	17.87	15.71	16.72	15.42	17.33	21.93	16.08	17.02	22.28	16.71	21.61	19.49
CCG	P	14.81	14.18	14.88	15.52	11.40	11.11	13.13	12.72	9.29	11.75	10.34	12.99	9.43	13.93	11.79	13.65
CCT	P	12.00	12.43	10.34	12.35	10.55	10.83	12.41	11.93	11.30	12.53	8.04	12.54	14.57	11.84	13.75	17.54
CAA	Q	21.40	20.35	23.15	20.91	20.26	21.67	20.32	23.06	19.84	21.93	21.83	20.15	15.42	22.63	27.51	27.29
CAG	Q	13.61	13.63	11.99	15.84	14.13	13.54	14.38	13.20	12.56	16.45	22.40	17.91	16.28	15.32	11.79	15.60
AGA	R	18.32	16.48	12.82	14.57	14.64	14.63	16.18	16.22	14.07	19.58	14.93	14.33	17.14	18.80	19.65	15.60
AGG	R	15.44	15.75	18.60	16.16	15.15	15.44	15.46	14.79	15.32	16.45	14.93	16.57	18.00	15.32	23.58	13.65
CGA	R	16.56	14.55	14.47	11.72	10.89	12.19	13.84	12.09	12.56	13.31	9.77	13.88	15.42	9.75	3.93	0.00
CGC	R	10.25	8.84	8.68	9.82	9.53	8.94	9.35	8.59	7.79	8.61	9.77	11.20	6.00	9.75	13.75	11.70
CGG	R	11.79	10.04	7.03	8.24	8.34	7.86	10.25	8.75	8.04	7.83	6.32	9.40	6.86	8.36	5.89	9.75
CGT	R	11.16	9.95	7.44	5.07	6.64	8.40	8.63	9.54	6.53	3.13	5.17	9.40	7.71	9.05	13.75	15.60
AGC	S	12.42	11.51	11.99	11.09	11.23	10.29	9.35	10.65	9.80	9.40	8.04	11.64	5.14	11.14	11.79	13.65
AGT	S	14.25	12.16	14.06	14.25	11.92	12.19	11.87	12.88	13.06	8.61	10.91	9.85	6.86	8.36	13.75	19.49
TCA	S	16.63	14.92	12.40	10.45	12.77	10.56	13.13	13.20	12.31	7.05	6.89	11.64	6.86	11.84	11.79	13.65
TCC	S	13.47	12.80	13.64	12.35	11.06	11.38	12.77	11.93	11.81	17.23	15.51	9.85	10.28	11.84	25.54	19.49
TCG	S	15.44	15.47	13.64	14.57	13.11	14.09	14.56	15.11	12.31	13.31	13.79	15.67	12.85	16.37	17.68	9.75
TCT	S	12.07	11.79	11.16	8.55	8.68	7.31	9.35	10.97	9.80	10.18	9.19	11.20	11.14	9.40	17.68	3.90
ACA	T	19.86	18.14	15.71	14.57	15.15	14.36	16.36	15.11	17.33	13.31	13.21	13.44	12.00	13.93	15.72	15.60
ACC	T	16.07	16.30	15.71	13.62	15.15	12.73	14.38	14.95	12.56	11.75	12.64	11.64	12.85	13.58	17.68	11.70
ACG	T	17.05	14.83	15.30	13.62	11.92	11.65	12.59	13.20	11.30	10.18	17.23	10.75	12.00	9.75	9.82	7.80
ACT	T	13.40	13.17	10.34	10.77	10.72	11.11	11.87	12.40	13.06	10.96	8.04	11.64	12.00	11.84	5.89	17.54
GTA	V	9.82	10.04	8.68	7.29	9.19	9.48	9.17	8.75	10.30	9.40	12.06	8.96	10.28	11.14	13.75	7.80
GTC	V	12.84	13.45	15.30	16.47	16.85	18.15	15.28	16.54	16.08	14.10	14.36	17.02	14.57	17.06	21.61	33.14
GTG	V	14.67	14.18	19.84	18.06	15.49	15.71	16.54	15.11	16.58	15.66	20.68	14.78	20.57	14.62	13.75	11.70
GTT	V	17.19	17.13	15.71	16.16	18.89	17.34	16.36	16.86	16.58	13.31	17.81	15.67	14.57	14.97	13.75	21.44
TGG	W	19.58	19.16	16.12	16.79	16.51	14.09	17.44	15.58	17.33	19.58	16.08	13.44	15.42	15.67	7.86	11.70
TAC	Y	13.75	15.66	23.15	18.69	20.94	21.67	17.44	17.65	17.33	20.36	18.96	18.81	23.14	18.11	25.54	25.34
TAT	Y	13.40	14.00	13.64	14.89	16.85	11.92	14.38	14.15	18.59	17.23	10.34	13.44	14.57	12.54	11.79	9.75

Table S5. Search commands used to retrieve the available RefSeq transcript files for each taxon on the *T. castaneum* phylogenetic scale of life. Each retrieved file corresponds to the RefSeq transcripts of a unique species.

Taxon	Search commands	Retrieved files
Coleoptera	"Coleoptera"[Organism] AND ("latest refseq"[filter] AND all[filter] NOT anomalous[filter])	6
Insecta	"Insecta"[Organism] NOT "Coleoptera"[Organism] AND "latest refseq"[filter]	33
Hexapoda	"Hexapoda"[Organism] NOT "Insecta"[Organism] AND "latest refseq"[filter]	1
Pancrustacea	"Pancrustacea"[Organism] NOT "Hexapoda"[Organism] AND "latest refseq"[filter]	2
Arthropoda	"Arthropoda"[Organism] NOT "Pancrustacea"[Organism] AND "latest refseq"[filter]	8
Ecdysozoa	"Ecdysozoa"[Organism] NOT "Arthropoda"[Organism] AND "latest refseq"[filter]	7
Protostomia	"Protostomia"[Organism] NOT "Ecdysozoa"[Organism] AND "latest refseq"[filter]	14
Eubilateria	"Deuterostomia"[Organism] AND "latest refseq"[filter]	129
Neuralia	"Cnidaria"[Organism] AND "latest refseq"[filter]	6
Eumetazoa	"Eumetazoa"[Organism] NOT "Cnidaria"[Organism] NOT "Bilateria"[Organism]	1
Metazoa	"Metazoa"[Organism] NOT "Eumetazoa"[Organism] AND (latest[filter] AND all[filter] NOT anomalous[filter])	1
Opisthokonta	"Opisthokonta"[Organism] NOT "Metazoa"[Organism] AND (latest[filter] AND all[filter] NOT anomalous[filter])	70
Amorphea	"Amoebozoa"[Organism] AND (latest[filter] AND all[filter] NOT anomalous[filter])	11
Eukarya	"Eukaryota"[Organism] NOT "Amoebozoa"[Organism] NOT "Opisthokonta"[Organism] AND (latest[filter] AND all[filter] NOT anomalous[filter])	148
	**Total**	**437**

Table S6. Search commands used to retrieve the available RefSeq transcript files for Archaea and Bacteria. Each retrieved file corresponds to the RefSeq genome of a unique species.

Taxon	Search commands	Retrieved files
Archaea	("Archaea"[Organism] OR Archaea[All Fields]) AND ("latest refseq"[filter] AND all[filter] NOT anomalous[filter])	740
Bacteria	("Bacteria"[Organism] OR Bacteria[All Fields]) AND ("latest refseq"[filter] AND "complete genome"[filter] AND "representative genome"[filter] AND all[filter] NOT anomalous[filter])	1,545
	Total	2,285
